# Age, primary symptoms, and genotype characteristics of norovirus outbreaks in Shanghai schools in 2017

**DOI:** 10.1038/s41598-018-33724-0

**Published:** 2018-10-15

**Authors:** Yuanping Wang, Lipeng Hao, Lifeng Pan, Caoyi Xue, Qing Liu, Xuetao Zhao, Weiping Zhu

**Affiliations:** 1 Center for Disease Control and Prevention of Pudong, 3039 Zhangyang Road, Pudong District, Shanghai, 200136 China; 2 Center for Disease Control and Prevention of Xuhui, 50 Yongchuan Road, Xuhui District, Shanghai, 200237 China

## Abstract

Sixty norovirus outbreaks that occurred in Pudong District, Shanghai in 2017 and affected 959 people were summarised. Of the outbreaks, 29 (48.3%), 27 (45.0%), and 4 (6.7%) occurred in kindergartens, primary schools, and middle schools, respectively. Although the total number of outbreaks peaked in March (13/60, 21.7%), outbreaks in kindergartens and primary schools peaked in April (6/29, 20.7%) and March (8/27, 29.6%), respectively. Primary schools had the highest median number of cases per outbreak (19) and the highest proportion of cases (54.6%). The male-to-female case ratio differed among school classifications, with the highest male case ratio (69.2%) occurring in middle schools. Primary symptoms also differed across the school classifications. Molecular virology analysis showed that a single viral strain caused each outbreak at each school. In turn, 50.6, 28.8, and 20.6% of cases were infected by GII.4, GII.2, and GII.17, respectively. Vomiting was seen in 98.2, 97.3, and 88.6% of the subjects infected with noroviruses GII.17, GII.4, and GII.2, respectively, and nausea in 73.6, 43.9, and 39.0%. In conclusion, noroviruses mainly affect primary school and kindergarten students. GII.4, GII.2, and GII.17 are the main epidemic strains in the local area, and the primary symptoms differed by age and genotype.

## Introduction

Noroviruses, previously known as Norwalk viruses, form a group of genetically diverse, positive-stranded non-enveloped RNA viruses belonging to the genus *Norovirus*, family Caliciviridae^1,2^. Their linear RNA genomes contain three open reading frames (ORFs)^3,4^. ORF1 encodes a large polyprotein, which is further cleaved by a viral protease into six non-structural proteins, including the viral polymerase^1–4^. ORF2 and ORF3 encode the major and minor capsid proteins VP1 and VP2, respectively^1–4^. Norovirus is a well-described cause of epidemic gastroenteritis across all age groups^2,5^. The U.S. Centres for Disease Control and Prevention (CDC) estimates that noroviruses are responsible for up to 60% of known-cause acute gastroenteritis cases^2,6^. The faecal–oral route is the main transmission mode, followed by other modalities, such as transmission via aerosolised viral particles in vomitus and through food, water, environmental contamination, and person-to-person^1–6^.

Students are frequently implicated in acute gastrointestinal outbreaks due to the concentrated population and close living environment in schools^7^. In China, outbreaks of infectious diarrhoeal disease also occur mainly in schools^8^. A systematic review and meta-analysis of data extracted from studies covering 85 outbreaks occurring from 1987 to 2014 showed that *Shigella*, pathogenic *Escherichia coli*, and norovirus were the most common pathogens to cause epidemic acute gastroenteritis in China^9^.

Thus far, complete data for whole-year norovirus outbreaks, regarding school type, season, primary symptoms, and genotype characteristics, remain rare in China. Shanghai is the largest city on the east coast of central China, with a total population of approximately 25 million. The Pudong District is located in eastern Shanghai with a population of 5.5 million, comprising 22.0% of the total population. In this report, we summarise 60 norovirus outbreaks that occurred in schools located in the Pudong District in 2017.

## Materials and Methods

### Ethics issues

This study was conducted in accordance with the World Medical Association Declaration of Helsinki and was approved by the Internal Review Board of the Centre for Disease Control and Prevention of Pudong District, Shanghai. Written informed consent was obtained according to the guidelines of the National Ethics Regulation Committee.

### Where, when, and who of outbreaks

Noroviruses cause inflammation of the stomach or intestines called acute gastroenteritis. The incubation period ranges from 12 to 72 h^10^. In this report, norovirus causing acute gastroenteritis was defined as sudden-onset vomiting or diarrhoea (*i*.*e*., abrupt-onset three or more loose or liquid stools above baseline in a 24-h period), with or without accompanying nausea, fever, or abdominal pain. An outbreak is defined as two or more cases with symptoms clustered in time and space.

This report summarises all norovirus outbreaks that occurred in kindergartens, primary schools, middle schools, and high schools in the Pudong District of Shanghai between January 1, 2017 and December 12, 2017. Norovirus outbreaks occurred in 29 kindergarten schools (students aged 4–6 years), 27 primary schools (students aged 7–11 years) and 4 middle schools (students aged 12–14 years) in this area in 2017. According to the relevant laws and regulations of the health authorities of China and Shanghai municipality, the sudden increase in the incidence of a disease or condition is reported to the local CDC. Infectious diarrhoeal disease is a key monitoring disease in schools. Each school reports students with diarrhoea, nausea, vomiting, abdominal pain, headache, and fever and/or chills, and the local CDC then carries out a field investigation. Investigation of the transmission source and route includes the collection of samples using rectal swabs of vomit, faeces, food, and water, and from the surfaces of public facilities. Environmental surface sampling was performed following the Guidelines for Environmental Infection Control in Health-Care Facilities (2003) recommended by the US CDC^11^. Briefly, the surfaces of classroom and bathroom doorknobs, faucets, desks, and chairs were lightly moistened with 1× phosphate-buffered saline (PBS; pH 7.4) containing 0.01% Tween™ 20 Surfact-Amps™ Detergent Solution (Thermo Fisher, Shanghai, China); cotton swabs were rolled across the moistened area quickly and rinsed in a 1.5-mL collection tube (Biovisualab, Shanghai, China) containing 0.5 mL PBS; all samples were kept on ice and submitted for pathogen detection within 6 h.

Investigation of the health management systems of schools includes consideration of vomitus disinfection, timely isolation, quality of daily disinfection, whether the sanitation facilities are adequate and reasonable, and management of public drinking water. All the parameters were presented as binary variables and recorded by a questionnaire.

### Data collection

Age, sex, initial symptoms, symptoms of acute gastroenteritis, histories of ingesting potentially contaminated food (expired food and spoiled food) and water (expired bottled water and unboiled water), contact with diarrhoea patients, travel 1 week before the onset of diarrhoea, and the administration of antibiotics were recorded using a questionnaire survey. The school, location, case number, number of student cases, number of employee cases, reporting date, data of the first case, data of the last case, and sampling number, as well as the above data from the field investigation and subsequent molecular virological analysis, were recorded.

### Norovirus detection

Faeces and vomit specimens were prepared as 10% (w/v) suspensions in distilled water and then centrifuged for 10 min at 10,000 × *g* in a 1.5-mL collection tube (Biovisualab) to remove any debris. The environmental surface samples were also centrifuged for 10 min at 10,000 × *g* in a 1.5-mL collection tube (Biovisualab) before viral RNA extraction. All centrifugation processes were carried out in a Thermo Scientific™ Sorvall™ Legend™ Micro 21 Microcentrifuge (Thermo Fisher Scientific, Shanghai, China) at 4 °C.

Viral RNA was extracted from the 140 μL suspensions using the QIAamp Viral RNA Mini Kit (QIAGEN, Venlo, Limburg, the Netherlands) according to the manufacturer’s instructions. Viral RNA was eluted with 60 μL RNase-free water containing 0.04% sodium azide (buffer AVE) and stored at −80 °C until polymerase chain reaction detection.

Real-time reverse-transcription polymerase chain reaction (RT-PCR) was used for the initial sample testing for noroviruses according to the method we described previously^12^. VP1 sequences were further amplified from real-time RT-PCR-positive samples. The primer pairs and probes used for sample screening were: forward, 5′-CARGARBCNATGTTYAGRTGGATGAG-3′; reverse, 5′-TCGACGCCATCTTCATTCACA-3′; and probe, 5′-TGGGAGGGCGATCGCAATCT-3′. The fragment encoding VP1 was amplified and sequenced using the forward primer 5′-CNTGGGAGGGCGATCGCAA-3′ and reverse primer 5′-CCRCCNGCATRHCCRTTRTACAT-3′. The expected PCR product size was 343 bp^12^.

The sequences of the VP1 regions were input into the Basic Local Alignment Search Tool (BLAST, http://blast.ncbi.nlm.nih.gov/Blast.cgi), and the genogroup and genotype definition were decided according to the “Sequences producing significant alignments of 100 Blast Hits on the Query Sequence.” Alternatively, the genogroup and genotypes were determined by the Norovirus Typing Tool Version 2.0 (https://www.rivm.nl/mpf/typingtool/norovirus/how-to-use). The correlation among all viral sequences was inferred using the maximum likelihood method based on the JTT matrix-based model. All sequence analyses were performed using MEGA7^13,14^.

### Statistical analysis

Categorical data are presented as frequencies with percentages; continuous variables are presented as means ± standard deviations or medians and upper and lower quartiles. Differences among groups were examined using Fisher’s exact probability test, the chi-square test or one-way analysis of variance, according to the characteristics of data distribution. *P* < 0.05 was considered to indicate statistical significance.

## Results

### Summary of outbreaks

In 2017, 60 outbreaks occurred in 29 (48.3%) kindergarten schools, 27 (45.0%) primary schools, and 4 (6.7%) middle schools; no outbreak was reported in high schools or other settings. There were 949 students and 10 employees in the schools with norovirus infections; the overall infection rate and median attack rate were 2.1% and 2.7%, respectively. The percentages of affected males and females were 52.1% and 47.9%, respectively. The average duration between the first and last cases was 3.5 ± 1.9 days. The proportions of cases with vomiting, nausea, abdominal pain, fever, and diarrhoea ranged from 95.3% to 10.4% (Table 1). In addition, 1.1% (6/542) of samples from the surfaces of public facilities were positive for noroviruses. Investigation of the health management systems of schools revealed incomplete disinfection of vomitus, lack of timely isolation and incomplete daily disinfection in 73.3, 61.7, and 51.7% of schools, respectively; these deficiencies might promote the transmission of noroviruses.

**Table 1:** Summary of the outbreaks.

Schools involved	
Total number of schools	60
Basic characteristics of the epidemics	
Total number of students and employees	45,878
Total cases	959 (2.1%)
Median attack rate	2.68%
Total student cases	949 (99.0%)
Total employee cases	10 (1.0%)
Male cases	500 (52.1%)
Female cases	459 (47.9%)
Interval between the first and last cases in days	3.5 ± 1.9
Primary symptoms	
Cases with vomiting	914 (95.3%)
Cases with nausea	444 (46.3%)
Cases with abdominal pain	279 (29.1%)
Cases with fever	160 (16.7%)
Cases with diarrhoea	100 (10.4%)
Investigation of the transmission source and route	
Samples of the transmission routes; positive/total samples	6/542 (1.1%)
Inadequacies in disease control and prevention	
Incomplete disinfection of vomitus	44/60 (73.3%)
Lack of timely isolation	37/60 (61.7%)
Incomplete daily disinfection	31/60 (51.7%)
Lack of sanitary facilities, such as hand washing stations	9 (15.0%)
Mismanagement of public drinking water	1 (1.7%)

Categorical data are presented as frequencies with percentages; intervals between the first and last cases are presented as mean ± standard deviation. The overall rate was calculated by dividing the total cases by the total number of students and employees (959/45,878 = 0.021). The other rates were calculated by dividing each frequency by the appropriate denominator.

### Outbreaks distributed by month

**Figure 1:**
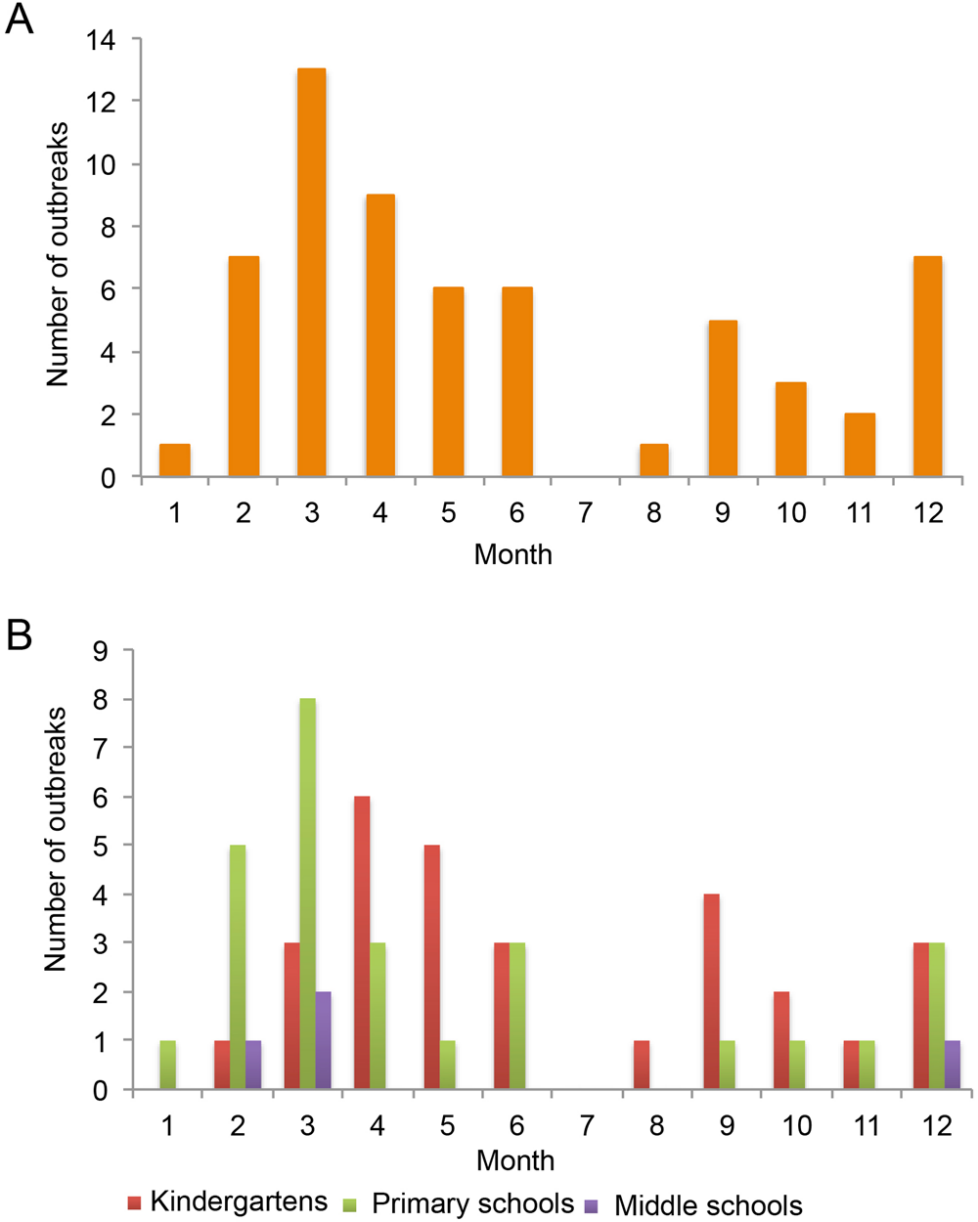
Outbreaks distributed by month. Outbreaks are plotted along the temporal axis. The number of outbreaks was (**A**) plotted by month and (**B**) further grouped by school types.

When the outbreaks were plotted along a temporal axis, most occurred in the first half of the year, with a peak in March (Fig. 1A). When these data were further grouped by school type, the epidemic trend differed among types (Fig. 1B). The outbreaks in kindergartens and primary schools peaked in April (six outbreaks) and March (eight outbreaks), respectively; whereas only four outbreaks occurred in middle schools and two of which were in March.

### Characteristics of norovirus outbreaks by school type

As shown in Table 2, the total number of cases and total student cases were concentrated mainly in the kindergartens and primary schools; the median numbers of cases per outbreak for kindergartens (12 cases) and middle schools (12 cases) were similar.

**Table 2:** Parameter distribution characteristics by agency.

	Kindergarten school	Primary school	Middle school	*P*
Schools involved				
Number of schools involved (n = 60)	29 (48.3%)	27 (45.0%)	4 (6.7%)	<*0*.*001*
Basic characteristics of the epidemics				
Total cases (n = 959)	383 (40.0%)	524 (54.6%)	52 (5.4%)	<*0*.*001*
Median cases per outbreak	12 (9.5, 17)	19 (14, 23)	12 (9.3, 17.8)	*0*.*007*
Total student cases (n = 949)	377 (39.7%)	521 (54.9%)	51 (5.4%)	<*0*.*001*
Total employee cases (n = 10)	6 (60.0%)	3 (30.0%)	1 (10.0%)	*0*.*038*
Male cases (n = 500)	180 (47.0%)	284 (54.2%)	36 (69.2%)	*0*.*004*
Female cases (n = 459)	203 (53.0%)	240 (45.8%)	16 (30.8%)	
Interval between the first and last cases in days	2.9 ± 1.7	4.0 ± 2.0	4.3 ± 1.3	*0*.*067*
Primary symptoms				
Cases with vomiting (n = 914)	355 (92.7%)	513 (97.9%)	46 (88.5%)	<0.001
Cases with nausea (n = 444)	152 (39.7%)	263 (50.2%)	29 (55.8%)	*0*.*003*
Cases with abdominal pain (n = 279)	145 (37.9%)	113 (21.6%)	21 (40.4%)	<*0*.*001*
Cases with fever (n = 160)	57 (14.9%)	100 (19.1%)	3 (5.8%)	*0*.*023*
Cases with diarrhoea (n = 100)	22 (5.7%)	61 (11.6%)	17 (32.7%)	<*0*.*001*
Investigation of the transmission source and route				
Samples of the transmission routes, positive/total samples	2/231 (0.9%)	4/277 (1.4%)	0/34 (0.0%)	*0*.*673*
Inadequacies in disease control and prevention				
Incomplete disinfection of vomitus (n = 44)	22 (75.9%)	21 (77.8%)	1 (25.0%)	*0*.*106*
Lack of timely isolation (n = 37)	17 (58.6%)	18 (66.7%)	2 (50.0%)	*0*.*462*
Incomplete daily disinfection (n = 31)	10 (34.5%)	19 (70.4%)	2 (50.0%)	*0*.*006*
Lack of sanitary facilities, such as hand washing stations (n = 9)	0 (0.0%)	6 (22.2%)	3 (75.0%)	<*0*.*001*
Mismanagement of public drinking water (n = 1)	0 (0.0%)	1 (3.7%)	0 (0.0%)	*0*.*517*

Categorical data are presented as frequencies with percentages; intervals between the first and last cases are presented as the mean ± standard deviation; median cases per outbreak are presented as the median (upper and lower quartiles). For categorical data, differences among groups were examined using the chi-square test or Fisher’s exact probability test when n ≤ 300. For continuous data, one-way analysis of variance (ANOVA) was used to determine the differences among groups.

A sex difference existed in the proportion of cases: in middle schools, the proportion of male cases (69.2%) was significantly higher than that of female cases (30.8%).

The primary symptoms also differed among the school types. The manifestations in kindergartens and primary schools, ordered from high to low occurrence, were vomiting, nausea, abdominal pain, fever, and diarrhoea. In the middle schools, the rates of cases with vomiting (88.5%) and fever (5.8%) were significantly lower than those in kindergartens and primary schools. Conversely, the rates of cases with nausea, abdominal pain, and diarrhoea were significantly higher than those in kindergartens and primary schools.

The deficiencies in the health management systems in schools varied among agencies. Lack of timely isolation was a common deficiency across all school types, with rates of 50.0% to 66.7%. Incomplete disinfection of vomitus was the dominant deficiency in kindergarten and primary schools, and the lack of sanitary facilities was a dominant deficiency in middle schools.

The proportion of adult cases (employees such as teachers and nursery governesses) was significantly higher in kindergartens (6/10) than in primary (3/10) and middle (1/10) schools.

### Characteristics of the norovirus outbreaks by genotype

Of the 60 outbreaks that occurred in 60 schools, viral VP1 sequencing of 9 to 20 specimens sampled from each school, corresponding to at least three independent individuals, was successfully completed in 50 schools (corresponding to 50 outbreaks). Of the 50 outbreaks with identified norovirus genotypes, 26 (52.0%), 14 (28.0%), 9 (18.0%), and 1 (2.0%) were caused by the GII.4, GII.2, GII.17, and GII.3 noroviruses, respectively. To determine the characteristics of the norovirus outbreaks by genotype, 49 outbreaks were regrouped as genotypes GII.2, GII.4, and GII.17 (the one GII.3 outbreak was not included in this analysis).

As shown in Table 3, GII.4 was the dominant epidemic strain across all school types; GII.2 and GII.17 had similar prevalence rates in middle and primary schools; GII.2 was the second common genotype to cause outbreaks in kindergartens. Although the prevalence rates of each viral genotype differed among school types, no statistical significance was observed for each school type.

**Table 3:** Parameter distribution characteristics by genotype.

	GII.2	GII.4	GII.17	*P*
Schools involved				
Number of schools involved (n = 49)	14 (28.0%)	26 (52.0%)	9 (18.0%)	<*0*.*001*
Middle school (n = 4)	1 (25.0%)	2 (50.0%)	1 (25.0%)	*1*.*000*
Primary school (n = 22)	4 (18.2%)	14 (63.6%)	4 (18.2%)	*0*.*326*
Kindergarten school (n = 23)	9 (39.1%)	10 (43.5%)	4 (17.4%)	*0*.*347*
Basic characteristics of the epidemics				
Total student cases (n = 782)	225 (28.8%)	396 (50.6%)	161 (20.6%)	<*0*.*001*
Total employee cases (n = 9)	3 (33.3%)	4 (44.4%)	2 (22.2%)	*0*.*716*
Male cases (n = 412)	107 (46.9%)	217 (54.3%)	88 (54.0%)	*0*.*181*
Female cases (n = 379)	121 (53.1%)	183 (45.7%)	75 (46.0%)	
Interval between the first and last cases in days	4.0 ± 2.6	3.4 ± 1.6	2.9 ± 1.7	*0*.*425*
Month of outbreaks	10 (8, 12)	4 (3, 5)	3 (3, 5)	*/*
Primary symptoms				
Cases with vomiting (n = 751)	202 (88.6%)	389 (97.3%)	160 (98.2%)	<*0*.*001*
Cases with nausea (n = 376)	100 (43.9%)	156 (39.0%)	120 (73.6%)	<*0*.*001*
Cases with abdominal pain (n = 210)	52 (22.8%)	105 (26.3%)	53 (32.5%)	*0*.*099*
Cases with fever (n = 130)	34 (14.9%)	66 (16.5%)	30 (18.4%)	*0*.*655*
Cases with diarrhoea (n = 79)	23 (10.1%)	39 (9.8%)	17 (10.4%)	*0*.*969*

Categorical data are presented as frequencies with percentages; the intervals between the first and last cases are presented as the mean ± standard deviation; months of outbreaks are presented as the median (upper and lower quartiles). For categorical data, differences among groups were examined using the chi-square test or Fisher’s exact probability test when n ≤ 300. For continuous data, one-way ANOVA was used to determine differences among groups.

The English in this document has been checked by at least two professional editors, both native speakers of English. For a certificate, please see: http://www.textcheck.com/certificate/huOrBz.

In order, 50.6, 28.8, and 0.6% of the student cases were infected by genotype GII.4, GII.2, and GII.17, respectively. No significant difference in gender susceptibility was observed among genotypes. In addition, no difference in disease course or adult case rates existed among the three genotypes. The median number of cases of GII.2, GII.4, and GII.17 was seen in October, April, and March, respectively (Table 3).

Interestingly, the primary symptoms, such as vomiting and nausea, showed genotype differences. The proportion of nausea was highest in GII.17 infection, although vomiting was the most common symptom and its incidence ranged from 88.6% in GII.2 infection to 98.2% in GII.17 infection. Although abdominal pain, fever, and diarrhoea were common symptoms, no genotype-specific rate difference was observed among the three genotypes (Table 3).

### Molecular phylogenetic characteristics of noroviruses

**Figure 2:**
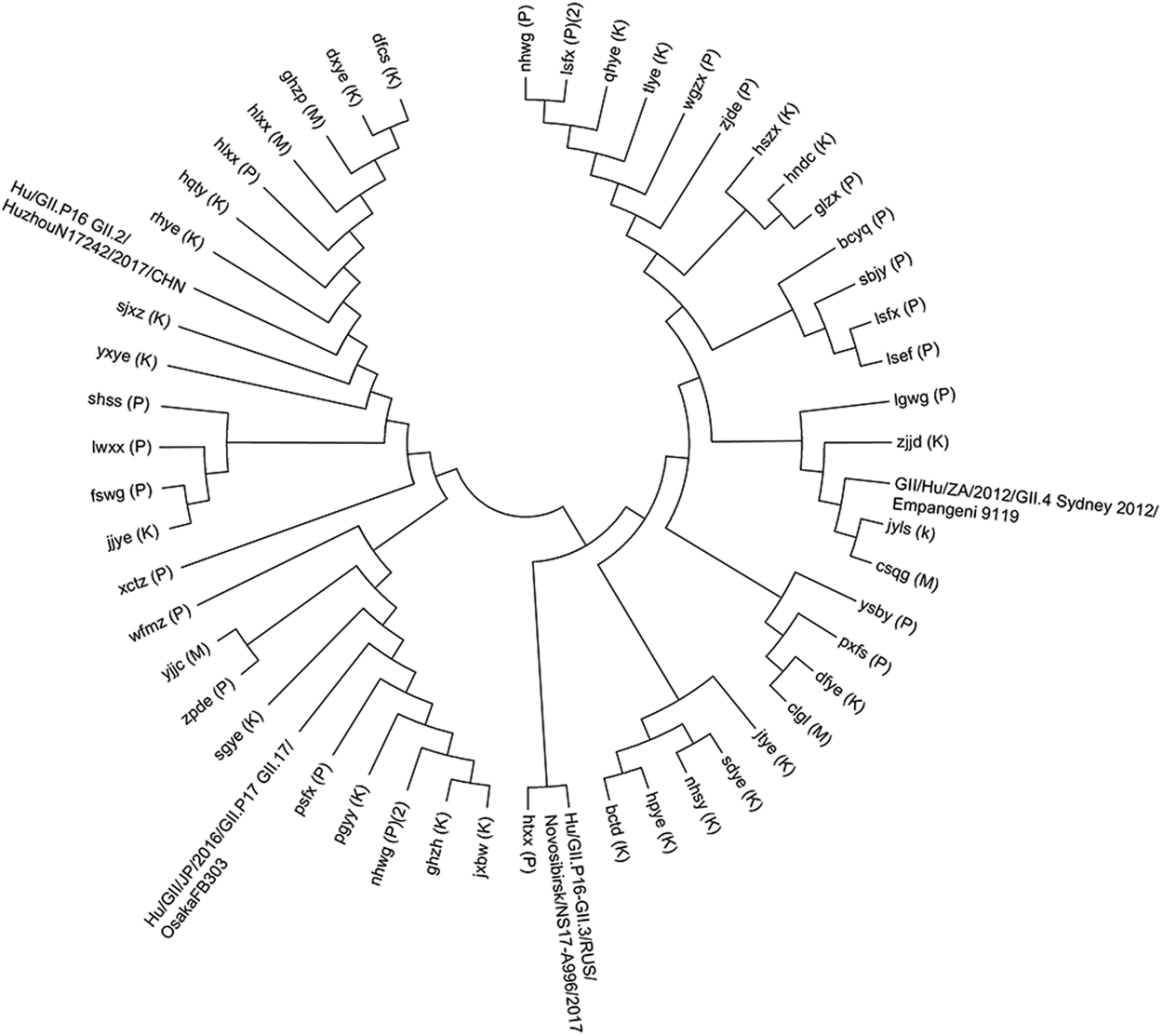
Molecular phylogenetic analysis by the maximum likelihood method. The evolutionary history was inferred using the maximum likelihood method based on the JTT matrix-based model. The tree with the highest log likelihood is shown. Initial tree(s) for the heuristic search were obtained automatically by applying Neighbour-Joining and BioNJ algorithms to a matrix of pairwise distances estimated using a JTT model and then selecting the topology with a superior log likelihood value. The tree is drawn to scale, with branch lengths measured as the number of substitutions per site. The analysis involved 54 sequences. To avoid ethical issues, all schools are represented by codes consisting of abbreviations of the full school names.

The viral sequences for each school were aligned to determine whether the outbreaks were caused by single or multiple strains. The alignment analysis showed 100% identity of the VP1 sequence obtained from each school; thus, one sequence from each school was selected to perform further viral correlation analysis among the schools. As shown in Fig. 2, for each genotype cluster, sub-clusters were formed for different agencies. Due to the high population density and complex community life relationships in Shanghai, we do not argue that these data can explain the virological linkage of outbreaks among schools. However, we are certain that GII.4, GII.2, and GII.17 can infect children in kindergarten, primary, and middle schools.

## Discussion

The illness caused by norovirus infection was initially described as “winter vomiting disease” in 1929 due to its seasonal predilection^1,2,15^. Our data suggested that age and viral genotype affect the seasonal predilection of norovirus outbreaks. In general, most outbreaks occurred in the first half of the year, with a peak in March. Outbreaks in kindergartens and primary schools peaked in April and March, respectively. The months in which the median number of GII.2, GII.4, and GII.17 outbreaks occurred were October, April, and March, respectively.

In 1968, an acute gastroenteritis outbreak occurred in an elementary school in Norwalk, OH, USA, and virologists identified the pathogen as norovirus^2,16^. Subsequently, nausea, vomiting, diarrhoea, and a low-grade fever were found to be the primary symptoms of norovirus infection^2,16^. Our data suggest that the primary symptoms were also associated with age and viral genotype: the manifestations in students in the kindergarten and primary schools, ordered from high to low proportion, were vomiting, nausea, abdominal pain, fever, and diarrhoea; among middle school students, the rates of vomiting (88.5%) and fever (5.8%) were significantly lower and the rates of cases with nausea, abdominal pain, and diarrhoea were significantly higher than among kindergarten and primary school students. In addition, the proportion of nausea was highest in GII.17-infected cases, although vomiting was the most common symptom, its concomitant probability ranged from 88.6% in GII.2-infected cases to 98.2% in GII.17-infected cases. Taken together, our data showed that diarrhoea is not a dominant symptom of norovirus infection and implied that age-specific host factors and genotype-specific viral factors play roles in the pathology of norovirus infection.

Human norovirus infections are caused, in decreasing order of frequency, by GII (mostly GII.4), GI, and, to a very limited extent, GIV genotypes^2,17–20^. However, this trend varies with time, place, and population in China. Since 2002, GII.4 viruses have been the most common genotype circulating in China^9,21,22^. However, GII.17, GII.P16/GII.2, and GII.6 variants have emerged in recent years and caused outbreaks in China^21,23,24^. The most prevalent genotypes in north and south China are GII.4, GII.1, and GII.3 and GII.4, GII.17, and GII.3, respectively^9^. The prevalent strains differ among populations due to differences in acquired immunity^21,25^. A systematic review has analysed the burden of acute gastroenteritis caused by norovirus infection in China since the year 2000^9^. The integrated data from more than 200 original reports showed that GII.4 (70.4%), GII.3 (13.5%), GII.17 (11.9%), and GII.1 (4.0%) were the top four prevalent strains in China^9^. The outbreaks in 26 (52.0%), 14 (28.0%), 9 (18.0%), and 1 (2.0%) of the schools in this report were caused by GII.4, GII.2, GII.17, and GII.3 norovirus infections, respectively. Although GII.4 and GII.17 were the first and third most common genotypes in our report, consistent with the above integrated data; GII.3 is rare and GII.2 emerged as the second most common genotype identified in our report. These differences might be explained by the fact that the integrated data included people of all ages from different regions of China, while our data only examined the epidemic characteristics of a specific population in a certain period of time in a local area.

*Limitations*: in this report, we did not trace the first case to identify the transmission source, as the first symptomatic case does not equate to the one originally excreting or being infected with the virus. On the other hand, although the government has been concentrating on improving water and food supplies in schools, sporadic waterborne or foodborne acute gastroenteritis cannot be extinct. The high population density and complex community life relationships in China weaken the significance of transmission source identification in outbreak control and prevention. As almost all were self-reported cases, age, sex, and personal psychosocial characteristics might affect the accuracy of self-reporting data and we could not exclude possible bias in case reporting. BLAST analysis showed that our sequences were highly similar to many reference sequences submitted from China and abroad. We could not decipher traceability because we lacked long-term background data on norovirus epidemics in China. In the present study, the viral genogroup and genotype were determined by partial VP1 sequences; thus, we could not fully study the recombinant noroviruses. Although this method is commonly used^26,27^, increasing evidence has supported the proposal to adopt a dual nomenclature using both ORF1 and VP1 sequences^28^ because recombination is common and the recognition of recombinant viruses may be relevant to their epidemiological characteristics^23,28–30^.

In conclusion, noroviruses GII.4, GII.2, GII.17, and GII.3 were in order the common genotypes in a local area. Noroviruses mainly affect primary school and kindergarten students and the primary symptoms differed by age and viral genotype.
